# Real-World Psilocybin Therapy for Treatment-Resistant Depression: A Retrospective Observational Study

**DOI:** 10.1016/j.lanepe.2026.101719

**Published:** 2026-06-01

**Authors:** Johannes Jungwirth, Samuel Westenhöfer, Helena D. Aicher, Barbora Provaznikova, Golo Kronenberg, Erich Seifritz, Susanne Prinz, Sebastian Olbrich

**Affiliations:** a Department of Adult Psychiatry and Psychotherapy, University Hospital of Psychiatry Zurich, University of Zurich, Switzerland; b Division of Clinical Psychology and Psychotherapy, Faculty of Psychology, University of Basel, Switzerland

**Keywords:** Psilocybin, Major depression, Depressive disorder, Treatment-resistant depression, Real-world, Psychotherapy, Psychedelic-assisted therapy, Compassionate-use

## Abstract

**Background:**

Psilocybin has demonstrated promising antidepressant effects in depression and treatment-resistant depression (TRD) in controlled clinical trials. However, its effectiveness and safety in real-world therapeutic settings remain largely unknown. Although psilocybin is not yet approved as an antidepressant treatment, Switzerland's unique legal framework allows its limited medical use for TRD. We aimed to examine antidepressant outcomes, safety, and feasibility of real-world psilocybin therapy for TRD, including repeated dosing sessions.

**Methods:**

We conducted a retrospective analysis of medical records from 19 TRD patients treated with psilocybin (20–35 mg) across one to four dosing sessions at the Psychiatric University Hospital Zurich. Depression severity was assessed using the Montgomery–Åsberg Depression Rating Scale (MADRS) and the Beck Depression Inventory II (BDI). Changes from baseline to interim and post-treatment were analysed, including response and remission.

**Findings:**

MADRS scores decreased from baseline (M = 30.78) to post-treatment (M = 19.89), with a large effect size (Hedges' g = 1.37, 95% CI [0.90, 1.84]). BDI scores also decreased (M = 32.33–M = 23.28), with a medium-to-large effect (Hedges’ g = .77, 95% CI [0.46, 1.09]). Response and remission rates were 33.3% and 22.2% (MADRS), and 27.8% and 27.8% (BDI). No serious adverse events were documented.

**Interpretation:**

This study provides some of the first evidence on psilocybin outside controlled trials. Psilocybin was associated with a clinically meaningful reduction in depressive symptoms, with response and remission rates below those reported in previous trials. Findings suggest feasibility of psilocybin in real-world TRD care and should be interpreted within the limitations of small sample size, retrospective uncontrolled design, heterogeneous treatment conditions, and concomitant psychopharmacology.

**Funding:**

This research did not receive any funding.

**Research in context**

**Evidence before this study**

Randomised controlled trials (RCTs) and recent meta-analyses show large short-term antidepressant effects of psilocybin compared to control conditions. Evidence derives mostly from studies in highly selected populations and under tightly standardised protocols. Generalisability to routine care and to complex treatment-resistant populations remains uncertain. Given the distinctive features of psychedelic interventions and related methodological challenges, real-world evidence is an important complement to RCTs. Real-world studies remain scarce, based on small, heterogeneous samples that often mix indications (e.g. cancer-related distress and major depression) or different substances and usually provide limited session-wise outcome data. We searched MEDLINE/PubMed, Embase, Cochrane Library, and ClinicalTrials.gov for reports on psilocybin from 2000 to Nov 30, 2025, without language restrictions, using combinations of terms for the intervention and indication (e.g., “psilocybin”, “psychedelic”, “depression”, “major depressive disorder”, “treatment-resistant depression”, “TRD”, “real-world”, “compassionate use”). We also screened reference lists of key articles, recent conference proceedings, and preprint servers for additional studies.

**Added value of this study**

This retrospective longitudinal observational analysis provides, to our knowledge, some of the first systematically collected real-world data on psilocybin treatment for treatment-resistant depression delivered under a regulated clinical framework. It reports clinician-rated and self-reported outcomes, uses mixed-effects modelling to examine session-wise change across up to four dosing sessions, and documents safety under routine conditions. We observed a reduction in depressive symptoms after the first dosing session that was largely maintained with subsequent sessions, no serious or sustained adverse events documented in the medical record, and response/remission rates that were lower than those in several controlled trials.

**Implications of all the available evidence**

Taken together, current evidence suggests that psilocybin has clinically relevant antidepressant effects in RCTs and those effects might be translatable to real-world treatment. Our findings may inform emerging regulated-access and compassionate-use programs by describing feasibility, treatment design, clinical outcome, and safety in routine care. They also support the development of prospective studies, to further investigate comparative outcomes, safety, standardisation, and predictors of response to enable patient stratification and efficient resource allocation.

## Introduction

Depressive disorders affect an estimated 330 million people globally and rank among the leading contributors to disease burden, with major depressive disorder (MDD) as the most prevalent subtype.^1^^,^^2^ Approximately half of the individuals with MDD who complete at least two antidepressant trials do not achieve remission.^3^ This group is commonly defined as suffering from treatment-resistant depression (TRD).^4^^,^^5^ Patients with TRD show a significantly lower quality of life and higher all-cause mortality, underscoring the urgent need for novel therapeutic options.^6^^,^^7^

In this context, psilocybin is emerging as a promising candidate. It primarily acts as a 5-HT2A receptor agonist, inducing dose-dependent alterations in cognition, perception, and affect.^8^ While the specific mechanisms underlying the antidepressant effect of psilocybin are not fully understood, they are likely to involve an interaction between neural plasticity, connectivity changes, and increased psychological flexibility.^9^ Several randomised controlled trials (RCTs) have demonstrated the efficacy of psilocybin in MDD, including TRD.^10^ Current data suggest that psilocybin is generally safe and well-tolerated when administered with psychological support in a clinical setting.^11^

Despite these encouraging findings, RCTs on psilocybin have faced methodological challenges. First, they are conducted under strict eligibility criteria. Patients suffering from severe treatment resistance, comorbidities, suicidality or specific medication histories are thus often excluded. Consequently, the generalisability of results to broader clinical populations remains uncertain.^12^ Second, the pronounced subjective effects of psilocybin make effective blinding difficult for both patients and raters, increasing the risk of bias in the treatment and control groups. This may inflate the estimation of psilocybin's efficacy.^13^ These limitations suggest that, despite their clear value, RCTs alone are insufficient to capture psilocybin's impact on clinical practice.

Another unresolved question is the durability of psilocybin's antidepressant effects and whether additional sessions may be clinically useful. While most trials rely on one or two dosing sessions, follow-up data suggest that symptoms may recur within months in some patients.^14^, ^15^, ^16^, ^17^ A solution to prevent these relapses may lie in multiple dosing sessions. Yet, evidence on multidose paradigms remains limited and mixed.^18^^,^^19^ Collectively, current research emphasises the necessity to examine psilocybin under flexible clinical conditions. Switzerland offers a unique legal framework to explore psilocybin treatment for TRD.^20^ From 2021, psilocybin can be prescribed under a so-called “limited medical use exemption” for several treatment-resistant indications issued by the Federal Office of Public Health.^21^ Treatment can be flexibly scheduled, and antidepressant medication can be continued. This is a key distinction to controlled trials, where medication was typically washed out and provides ideal conditions to explore the real-world feasibility of psilocybin treatment. To our knowledge, only two studies have been conducted in a comparable setting. The first used a sample of eight patients to explore the long-term effects of psilocybin on alleviating distress associated with life-threatening diseases in Canada.^22^ The second primarily examined the relationship between the acute subjective drug effects and the change in depression with both lysergic acid diethylamide and psilocybin.^23^

The present study describes the implementation and short-term outcomes of psilocybin therapy for TRD. Treatment was delivered under real-world clinical conditions, with medication management and dosing guided by shared decision-making. We retrospectively explore changes in depressive symptoms as well as the tolerability and feasibility of repeated psilocybin administrations. Session-wise analyses beyond the second session are descriptive, as re-dosing was clinically indicated rather than protocol-driven. By observing the course and feasibility of multiple psilocybin dosing sessions in a real-world setting, we hope to inform future trials and the development of clinical treatment plans, as regulatory approval processes are expected in the coming years.

## Methods

### Study design

This retrospective observational study was conducted at the University Hospital of Psychiatry Zurich, Switzerland. Data were extracted from electronic health records in 2025. Ethical approval was granted by the Cantonal Ethics Committee Zurich (BASEC-Nr. 2025-00559). All patients provided written consent for the use of anonymised clinical data.

### Participants

All patients underwent treatment at the Second-Opinion-Consultation-Service for Depression, a specialised outpatient consultation and treatment service for TRD patients. Patients were referred to the consultation service by external and internal psychiatrists, psychotherapists, or general practitioners. Participants were aged 27–67 years and treated between 02/2023 and 05/2025. All had been diagnosed with a treatment-resistant mild to severe depressive episode by trained psychiatrists according to ICD-10 criteria. To be categorised as treatment-resistant, patients were required to have undergone at least two antidepressant trials of adequate dose and duration in accordance with clinical guidelines, without reaching response or remission of depressive symptoms. Adequacy and adherence were verified through review of referral documentation, medication lists, and patient reports during the structured psychiatric assessment. Staging criteria were not systematically applied. Since comorbidities are often present in TRD, they did not lead to exclusion unless they were considered contraindications; see below. Active substance use disorder and personality disorder were not per se exclusion criteria, and no exclusions were attributable to either. By contrast, individuals with acute suicidality requiring crisis intervention were not offered psilocybin treatment. Patients were required to be in stable physical health and not show any contraindications. Exclusion criteria were a personal or first-degree family history of psychotic or bipolar disorders, arterial intracranial aneurysm on magnetic resonance imaging, clinically relevant cardiac arrhythmias or signs of recent cardiovascular events on electrocardiogram, epileptiform activity or increased seizure susceptibility on electroencephalogram, uncorrected hypertension, a history of stroke, intracerebral haemorrhage, or any aneurysm, increased intracranial pressure, inadequately treated hyperthyroidism, (planned) pregnancy, or breastfeeding. A summary of patient demographics and clinical characteristics is given in Table 1.

**Table 1:** Participant demographics and clinical characteristics.

Variable	Yes *n* (%)	Details
Demographics		
Age (years)		*N* = 19; *M* = 47.5, *SD* = 12.7, Min = 27.0, Max = 67.0
Sex	6 (31.6%)	Female 6 (31.6%)
		Male 13 (68.4%)
Region of origin		Europe 18 (94.7%)
		Outside Europe 1 (5.3%)
Prior psychedelic experience	5 (26.3%)	Psilocybin (2 (10.5%))
		Lysergic acid diethylamide (1 (5.3%))
		Unspecified (2 (10.5%))
Context of prior psychedelic experience		Medical setting (2 (10.5%))
		Recreational (3 (15.8%))
Duration of current depressive episode (at baseline in years)		≥15 (4 (21.1%))
		8–14 (3 (15.8%))
		3–7 (9 (47.4%))
		≤2 (3 (15.8%))
Clinical Characteristics		
MADRS baseline		N = 19; M = 31.3, SD = 9.1, Min = 15.0, Max = 43.0
BDI baseline		N = 19; M = 33, SD = 12.3, Min = 14.0, Max = 52.0
Comorbidities (at baseline)		Subthreshold features of personality disorder (6 (31.6%))
		Anxiety Disorders (5 (26.3%))
		Post-traumatic stress disorder (3 (15.8%))
		Attention-deficit hyperactivity disorder (2 (10.5%))
		Mental and behavioural disorders due to psychoactive substance use (2 (10.5%))
		Obsessive-compulsive disorder (2 (10.5%))
		Disorders of adult personality and behaviour (1 (5.3%))
		Bulimia nervosa (1 (5.3%))
		Pain disorder (1 (5.3%))
		Aspergers Syndrome (1 (5.3%))
Treatment History		
Antidepressant trials (at baseline)		*N* = 19; *M* = 5.0, *SD* = 2.8, Min = 2.0, Max = 14.0
Augmentation (at baseline)	6 (31.6%)	Atypical antipsychotic (5 (26.3%))
		Lithium (2 (10.5%))
Augmentation attempts (at baseline)		>2 (7 (36.8%))
		2 (3 (15.8%))
		1 (6 (31.6%))
		0 (3 (15.8%))
Prior esketamine treatment	7 (36.8%)	
Antidepressant medication (at baseline)	17 (89.5%)	SARI (7 (36.8%))
		SNRI (7 (36.8%))
		SSRI (6 (31.6%))
		MAO-Inhibitors, TCAs and others (3 (15.8%))
		SMS (2 (10.5%))
Other psychiatric medication (at baseline)		No other medication (12 (63.2%))
		Benzodiazepine (3 (15.8%))
		Anticonvulsant (2 (10.5%))
		Hypnotic (1 (5.3%))
		Typical antipsychotic (1 (5.3%))
Psychotherapy (at baseline)	15 (78.9%)	Cognitive behavioural therapy (11 (57.9%))
		Other (3 (15.8%))
		Psychoanalysis (3 (15.8%))
		Unknown/Missing (4 (21.1%))
Psilocybin treatment characteristics		
Psilocybin dose categories (instances N = 40)		20 mg (1 (2.5%))
		25 mg (27 (67.5%))
		30 mg (10 (25%))
		35 mg (2 (5%))
Number of sessions (instances N = 40)		1 Session (19 (100%))
		2 Sessions (13 (68.4%))
		3 Sessions (5 (26.3%))
		4 Sessions (3 (15.8%))
Total treatment duration		N = 19, M = 73.42, SD = 90.28 Min = 14, Max = 336

Notes. Values are n (percentage of all patients) unless otherwise specified. Continuous variables report N, M, SD, minimum (Min), and maximum (Max). Subthreshold features of personality disorder refer to clinically noted personality features that did not meet ICD-10 or DSM criteria for a personality disorder. Abbreviations: MADRS, Montgomery-Åsberg Depression Rating Scale; BDI, Beck Depression Inventory—II, SSRI, selective serotonin reuptake inhibitor; SNRI, serotonin-norepinephrine reuptake inhibitor; SARI, serotonin antagonist and reuptake inhibitor; SMS, serotonin modulator and stimulator; MAO-Inhibitors, Monoamine oxidase inhibitors; TCAs, Tricyclic antidepressants.

### Procedure

#### Treatment course and dosing

The treatment procedure assigned to each patient was based on availability and clinical judgement. Treatment was delivered in three pathways: inpatient group setting (n = 11), single inpatient setting (n = 7), and single outpatient setting (n = 1). With one exception, patients remained within their pathway; one planned group session was delivered as a single session due to a lack of other participants. Across pathways, patients received between one and four dosing sessions. The number of dosing sessions in the single settings was individually and dynamically planned and could be a few months apart. In the inpatient group setting, the number of dosing sessions was preplanned to two dosing sessions, two weeks apart, within a 4-week admission. Decisions regarding additional treatment sessions and specific dose were made collaboratively between clinicians and patients, considering existing evidence, clinical assessment, patient preferences, and perceived and expected benefit. Additional sessions were mainly considered for patients who demonstrated a partial response or non-sustained improvement. If no meaningful benefit was observed after an initial session, the treatment plan was re-evaluated before proceeding. Patients received an individualised dose of orally administered synthetic psilocybin, ranging from 20 to 35 mg. Psilocybin was obtained from Dr Hysek, Biel, Switzerland and formulated according to Good-Manufacturing-Practice in capsules containing 5 mg.

#### Psychotherapeutic framework

Psychological support followed a manual-guided, non-directive, psychedelic-informed psychotherapeutic approach. All patients received multiple preparation and integration sessions before and after dosing, delivered by specially trained clinicians. Clinicians were psychiatrists and clinical psychologists; five therapists were involved. Patients were typically assigned a primary therapist who remained involved across sessions. Preparation and integration sessions lasted approximately 60 min each. The number, timing, and detailed content of sessions are outlined below and in the supplement. Clinicians were guided by a specifically designed manual but were allowed to apply psychotherapeutic methods consistent with their professional training. The manual was informed by a study protocol of an earlier trial on site and contained structured guidance for preparation, dosing and integration.^16^ Training consisted of site-specific workshops and lectures to standardise core elements. Nursing staff also took part to ensure consistent support and safety procedures. Inpatient group patients received two additional group meetings before and after dosing; otherwise, support was similar across settings. Supervision and intervision of clinicians were held regularly. Beyond that, no formal fidelity monitoring was performed. While manual-guided, the approach was intentionally flexible and not strictly manualised; psychotherapy exposure is therefore a potential source of confounding.

#### Preparation and medication management

Before preparation, patients underwent psychiatric assessment, including biographical history, psychometric testing, and medical examinations. Patients then completed 2–4 individual preparatory sessions focused on establishing therapeutic rapport and managing expectations. Decisions regarding concomitant medication were guided by clinical judgement, patient preferences, and an interaction table,^24^ with pharmacology consultation when needed. Antidepressant medication was mostly continued to support clinical stability, and only paused on the dosing days. If continuation was not considered safe, medications were tapered beforehand and, in most cases, resumed afterwards. For a few patients, tapering reflected a personal treatment goal, which was supported when clinically appropriate. Medication with 5-HT2 receptor blocking properties was tapered/paused when clinically feasible at least three half-lives prior to the dosing session to minimise potential pharmacodynamic attenuation.

Concomitant medication status was structured as follows. Across treatment (patients = 19), two were without antidepressant medication, 11 continued the antidepressant (10 paused at dosing days; one remained once on dosing day), two changed, and four stopped antidepressant during the treatment course. Of these 19 patients, six received an atypical antipsychotic (three paused beforehand and resumed afterwards, and three stopped beforehand and did not restart), and two had lithium (one paused beforehand and resumed afterwards, and one stopped beforehand). Eight patients took mirtazapine or trazodone (all paused or stopped before dosing), two were on benzodiazepines, and four took benzodiazepines once around the dosing day.

More information on medication management is available in the supplement in Table S1 showing medication status and in Table S8 outlining how decisions regarding concomitant medication were informed.

#### Dosing session

Patients received psilocybin in a calm and specifically arranged room. The dosing session began at approximately 9:00 a.m. with oral administration of psilocybin and continued until the acute subjective effects had largely subsided, typically around 4:00 p.m. In the single setting, at least one specifically trained therapist was present during the whole dosing session. In the inpatient group setting, at least two therapists were present during the whole session. At the end of the dosing day, patients were given a questionnaire to assess adverse events (AEs).

#### After dosing session

Following dosing day, patients attended ≥2 integration meetings aimed at reflecting the psychedelic experience, discussing emerging insights, and supporting transfer to daily life.

Detailed information on preparation, dosing day, and integration, can be found in the supplement.

### Measures

#### Depression outcomes

The primary outcomes were scores on the Beck Depression Inventory II (BDI) and the Montgomery–Åsberg Depression Rating Scale (MADRS) after the final dosing session.^25^^,^^26^ MADRS scores were obtained through interviews conducted by trained clinicians, whereas BDI scores were completed by patients as self-reports. Assessments were collected during routine visits before, between, and after dosing sessions. Scores were extracted directly from scale entries and were not reconstructed from narrative clinical notes.

Secondary outcomes included response, defined as a ≥50% reduction from baseline, and remission, defined as a total score reduction to ≤13 on the BDI and to ≤12 on the MADRS.

#### Adverse events

AEs were a secondary outcome collected through a questionnaire containing 10 commonly occurring AEs during psilocybin treatment (Table 2). Questionnaires were not available for all dosing sessions. Beyond the questionnaire, serious and sustained AEs, including increasing suicidality, psychotic or manic symptoms, prolonged anxiety, and sensory abnormalities, were monitored in clinical procedures, documented in the medical record, and treated where necessary. These follow-up AEs after the dosing day were assessed less systematically than same-day AEs.

**Table 2:** Frequency of adverse events.

Adverse Event	Patients n (%)	Sessions n (%)
Fatigue	12 (63.2%)	20 (66.7%)
Headache	9 (47.4%)	16 (53.3%)
Difficulty concentrating	8 (42.1%)	11 (36.7%)
Tearfulness	7 (36.8%)	15 (50.0%)
Anxiety	7 (36.8%)	11 (36.7%)
Nausea	7 (36.8%)	8 (26.7%)
Impaired balance	5 (26.3%)	6 (20.0%)
Dizziness	5 (26.3%)	5 (16.7%)
None	4 (21.1%)	6 (20.0%)
Dry mouth	3 (15.8%)	5 (16.7%)
Palpitations	3 (15.8%)	3 (10.0%)

Note. N(patients) = 19; N(sessions) = 30; In the patients column every AE is only counted once for each patient.

### Statistical analysis

The primary objective of this study was to estimate changes in depressive symptoms in patients who underwent psilocybin treatment. All analyses were exploratory and focused on effect estimates and uncertainty, without significance testing. Baseline was defined as the closest measurement prior to the first dosing session. If BDI and MADRS assessments were not conducted on the same day, the two closest assessments were chosen.

#### Primary endpoint analyses

For all primary endpoint analyses, post-treatment was defined as the first available measurement within 42 days after the final dosing session. This window was chosen as a pragmatic way to capture early post-treatment outcomes without overly restricting sample size. This follow-up window should thus be interpreted as reflecting an early post-treatment status rather than longer-term follow-up. Primary endpoint analyses included all patients who had a baseline assessment and at least one eligible post-treatment assessment. Missing and excluded values were not imputed. To assess changes in depressive symptoms from baseline to post-treatment, the mean change was calculated. Uncertainty was expressed using t-based 95% confidence intervals (CIs). To determine the effect size of the within-person change, Hedges' g was calculated.^27^ Uncertainty around Hedges’ g was quantified using participant-level bootstrap resampling (2000 resamples), with bias-corrected and accelerated 95% CIs.

To account for a possible bias in assessment timing, since post-treatment scores were collected at different time points, a multiple linear regression model was fitted to predict post-treatment scores using baseline scores and the number of days since treatment as predictors. Additional covariate adjustment was avoided due to sample size constraints. Regression coefficients are reported with 95% CIs.

To assess whether group-level changes were clinically meaningful, remission (MADRS ≤12; BDI ≤13) and response (≥50% reduction) were calculated.

#### Changes in depressive symptoms across sessions

To assess changes across dosing sessions, a linear mixed-effects model was fitted.^28^ It was estimated using restricted maximum likelihood. No imputations of missing data were made. All available observations were used under a missing-at-random (MAR) assumption. However, because continuation to later sessions was likely influenced by several factors, including clinical course and motivation, the MAR assumption may not have been fully met, particularly for sessions three and four. Accordingly, estimates for sessions three and four were interpreted descriptively only. Degrees of freedom were approximated using Satterthwaite's method. The outcome variable for the linear mixed model was derived by selecting the first available score recorded within 42 days after each dosing. The model included sessions as a factor from baseline to session four as a fixed effect, and participants as random intercepts to account for baseline differences among individuals. Session contrasts, defined as change from baseline, were obtained from the linear mixed-effects model. Model-based estimated marginal means (EMMs) were computed for each dosing session. Both were reported with 95% CIs and interpreted descriptively.

The following sensitivity analyses were conducted to evaluate the robustness of the linear mixed-effects model. All analyses used the same estimation procedures and the same missing data approach as the primary model. First, an extended model using time since last dosing session as a covariate was fitted. Because baseline assessments occurred before any dosing, days since the last dosing session were set to 0 at baseline. Second, to evaluate the impact of the assessment window, the model was refitted using a more restrictive 14-day window. Third, to assess whether estimates were disproportionately influenced by patients undergoing three or more dosing sessions, a model with patients who underwent only the first two sessions was fitted.

#### Adverse events

AEs were summarised by type, first, based on how many participants exhibited them at least once, and second, on how many times they occurred in all dosing sessions. Serious and sustained AEs documented in medical records were screened for in all relevant clinical notes.

All analyses were conducted using R (version 4.5.0) and RStudio (version 2025.05.1 + 513). A full list of the key packages and a summary of assumptions tested can be found in the supplement.

### Role of the funding source

This research did not receive any funding.

## Results

**Fig. 1:**
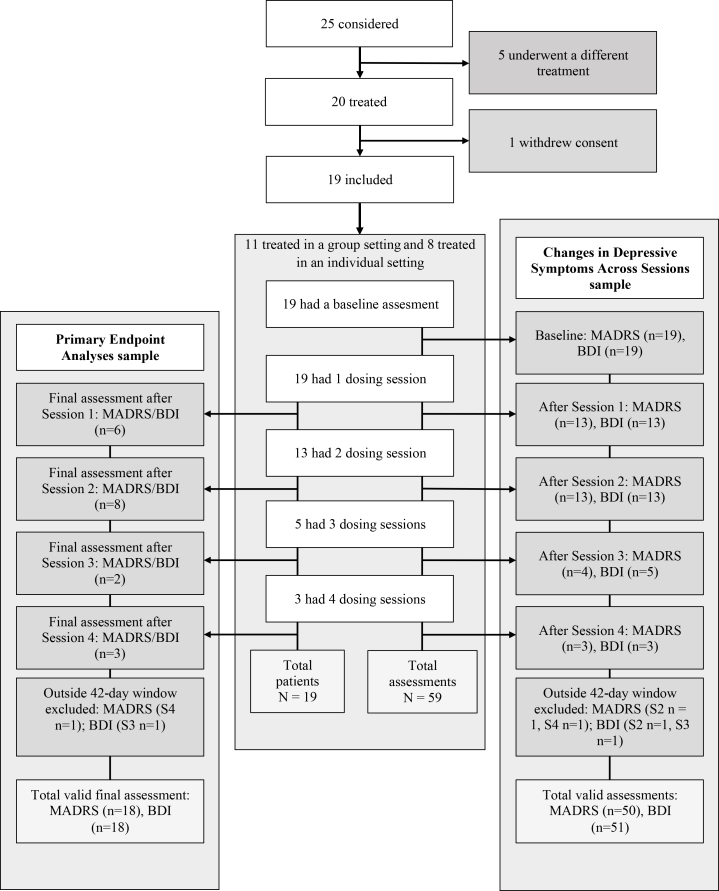
**Patient assembly and data used for analyses**. SX, session X.

Out of 25 patients considered for psilocybin treatment, 5 decided to pursue a different treatment, and 1 did not consent to research use of their data. This left 19 patients for the final sample who underwent at least one dosing session, 13 who underwent two sessions, 5 who underwent three sessions, and 3 who underwent four sessions, yielding a total of 40 sessions. Patient flow and the samples used for analyses are described in Fig. 1.

### Primary endpoint analyses

For the primary endpoint analyses, one participant did not have an assessment within 42 days post-treatment on the MADRS, and another did not have a post-treatment assessment within the 42-day window on the BDI. Both patients were thus excluded from the analysis, leading to a final n = 18 on each instrument.

**Fig. 2:**
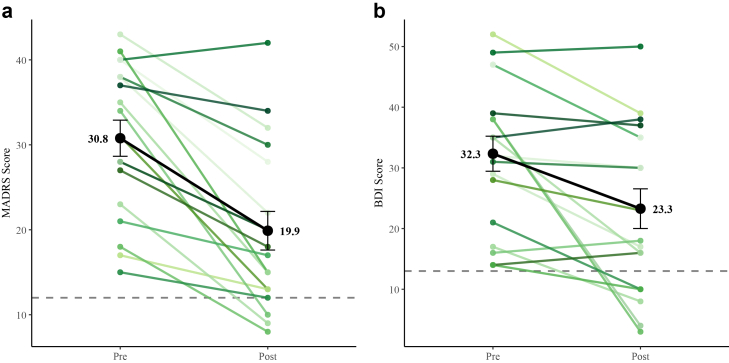
**a. Change in MADRS scores from pre-to post-treatment**. The black line and error bars indicate the group mean ± SE. The dashed line marks the remission threshold (MADRS ≤12). **b. Change in BDI scores from pre-to post-treatment**. The black line and error bars indicate the group mean ± SE. The dashed line marks the remission threshold (BDI ≤13).

To assess changes in depressive symptoms, mean difference, associated effect size and CIs were calculated. The mean MADRS score decreased from 30.78 (SD = 9.02) at baseline to 19.89 (SD = 9.65) after the last dosing session (Fig. 2a). The mean reduction was 10.89 points, 95% CI [7.10, 14.68], with a large to very large effect size (Hedges' g = 1.37, Bootstrapped 95% CI [0.90, 1.84]). The mean BDI score decreased from 32.33 (SD = 12.25) points at baseline to 23.28 (SD = 13.88) after the last dosing session (Fig. 2b). The mean reduction was 9.06 points, 95% CI [3.48, 14.63], with a medium to very large effect size (Hedges’ *g* = 0.77, Bootstrapped 95% CI [0.46, 1.09]). Out of 18 patients, 6 (33.3%) and 5 (27.8%) were classified as responders on the MADRS and BDI, respectively. The mean percentage reduction was 35.6% on the MADRS and 26.8% on the BDI. In total, 4 patients (22.2%) on the MADRS and 5 patients (27.8%) on the BDI met remission criteria at the end of the treatment period. These results indicate that patients improved across the treatment period on both clinician- and self-rated measures.

To assess whether follow-up timing biased MADRS and BDI post-treatment scores, a sensitivity multiple regression analysis using baseline scores and the time in days since the patients’ final dosing session to predict post-treatment scores was conducted. The follow-up assessment timing indicated that the primary endpoint analyses mainly reflected early post-treatment outcomes, although follow-up timing varied, particularly for MADRS (median 4.5 days, IQR 2.0–25.25) compared to the BDI (2 days, IQR 1.0–4.75). The adjusted association between days since the final dosing session and MADRS post-treatment scores was β = 1.08 (95% CI [−0.67 to 2.84]) points per seven days. On the BDI, the adjusted association between days since the final dosing session and post-treatment scores was β = 1.54 (95% CI [−1.87 to 4.95]) points per seven days. Estimates across instruments were small, and CIs included slight decreases as well as moderate increases over seven days; overall, these results are not suggestive of substantial time-related bias. Full multiple regression results are presented in Table S2 of the supplement.

### Changes in depressive symptoms across sessions

**Fig. 3:**
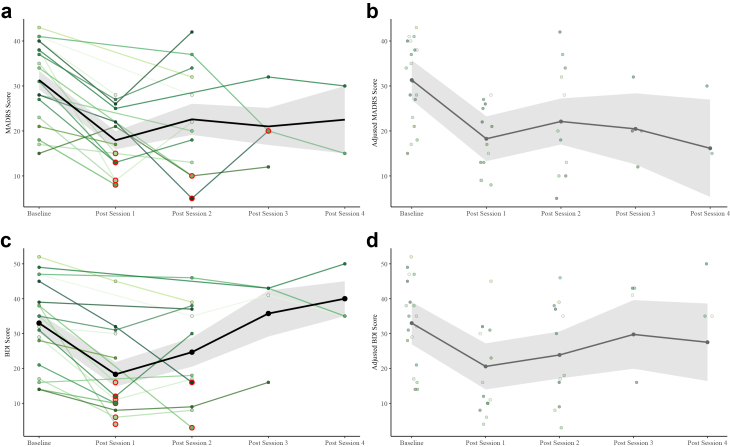
**a. Trajectory of clinician-rated depression severity (MADRS) across sessions**. The black line and translucent band indicate the group mean ± SE. Red-outlined dots mark the sessions at which a ≥50% reduction from baseline was reached for the first time. **b. Estimated marginal means of clinician-rated depression severity (MADRS) across sessions**. The line and points show model-adjusted means; the shaded band shows the 95% CI. Green coloured dots represent participant scores. **c. Trajectory of self-rated depression severity (BDI) across sessions**. The black line and translucent band indicate the group mean ± SE. Red-outlined dots mark the sessions at which a ≥50% reduction from baseline was reached for the first time. **d. Estimated marginal means of self-rated depression severity (BDI) across sessions**. The line and points show model-adjusted means; the shaded band shows the 95% CI. Green coloured dots represent participant scores.

To assess changes across sessions, a linear mixed-effects model was fitted. After removing all assessments outside the 42-day window, 50 remained on the MADRS and 51 on the BDI. At baseline, the mean MADRS score was 31.3 (SD = 9.1) and the mean BDI score was 33 (SD = 12.3). After session one, the mean scores were 17.9 (SD = 7.31) on the MADRS and 18.3 (SD = 12.6) on the BDI; after session two, 22.6 (SD = 11.98) and 24.8 (SD = 14.4); after session three, 21 (SD = 8.3) and 35.8 (SD = 13.2). On the MADRS, two patients were assessed after session four (M = 22.5, SD = 10.61), and on the BDI, three patients (*M* = 40, *SD* = 8.7). Fig. 3a and c show the trajectory of depression scores across sessions.

The model-estimated baseline mean for the MADRS was 31.3 (95% CI [26.9, 35.7]). At session one, the estimated mean was 18.3 (95% CI [13.3, 23.2]), corresponding to an estimated mean reduction of 13.1 points (95% CI [8.3, 17.8]). After session two, the estimated mean was 22.1 points (95% CI [17.0, 27.2]); this remained a mean reduction from baseline of 9.2 (95% CI [4.4, 14.2]). After session three, the estimated mean was 20.5 (95% CI [12.5, 28.4]), with an estimated mean reduction from baseline of 10.9 points (95% CI [3.4, 18.5]). After session four, the estimated mean was 16.1 (95% CI [5.2, 27.1]), representing an estimated mean reduction of 15.17 (95% CI [4.6, 25.8]). A visual representation of the estimated means after each session with the corresponding 95% CI can be found in Fig. 3b. The intraclass correlations (ICCs) showed that 54% of the variance was between patients, warranting random intercepts.

On the BDI, the model estimated a baseline mean of 33 (95% CI [26.6, 38.9]). After session one, the estimated mean was 20.6 (95% CI [14.0, 27.2]), with an estimated mean reduction of 12.42 (95% CI [7.2, 18.0]). After session two, the estimated mean was 23.9 (95% CI [17.1, 30.6]), with an estimated mean reduction from baseline of 9.1 (95% CI [3.1, 14.62]). After session three, the estimated mean was 29.8 (95% CI [19.9, 39.6]), representing an estimated mean reduction of 3.3 (95% CI [−5.6, 12.2]). After session four, the estimated mean was 27.5 (95% CI [16.4, 38.6]), representing a mean reduction of 5.49 (95% CI [−5.3, 15.5]). A visual representation of the estimated means after each session with corresponding 95% CI can be found in Fig. 3d. The ICC showed that 68% of the variance was between patients, indicating that random intercepts were warranted.

In summary, estimates indicate an early decrease in depressive symptoms across instruments following session one. While these levels were largely maintained on the MADRS, on the BDI, estimates shifted towards baseline levels in sessions three and four. This shift was accompanied by a widening of CIs, reflecting an increase in uncertainty associated with sparse data at sessions three and four. The linear mixed model results for both instruments are provided in Table S3a and b.

To assess the robustness of linear mixed-effects model results, three sensitivity models were fitted; the corresponding EMMs are shown in Table 3. Adding days since the last dosing session, restricting the assessment window to 14 days, and excluding patients who underwent more than two dosing sessions, only marginally affect estimates, while 95% confidence intervals overlapped substantially, suggesting limited evidence that the model estimates were meaningfully biased by follow-up timing or patients who underwent more than two dosing sessions. The timing of follow-up assessments on both instruments indicated that these analyses primarily captured early post-session effects (MADRS: median = 5 days, IQR: 1.5–13.0; BDI median = 2 days, IQR: 1.0–5.5). Full linear mixed-effects model results can be found in Tables S4 through S6.

**Table 3:** Estimated marginal means by session across sensitivity models.

Sensitivity model (sample)	Baseline 95% CI	Post S1 95% CI	Post S2 95% CI	Post S3 95% CI	Post S4 95% CI
**MADRS**					
Sensitivity 1 (n1 = 19, n2 = 50)	31.3 [27.1, 35.5]	15.5 [10.1, 20.8]	18.2 [12.2, 24.2]	19.0 [11.3, 26.7]	16.6 [6.18, 27.0]
Sensitivity 2 (n1 = 19, n2 = 44)	31.3 [27.2, 35.4]	18.2 [13.2, 23.2]	18.3 [12.7, 24.0]	21.1 [13.4, 28.8]	18.3 [7.6, 29.1]
Sensitivity 3 (n1 = 14, n2 = 32)	30.9 [25.8, 35.9]	15.8 [10.4,21.2]	22.8 [17.0, 28.5]		
**BDI**					
Sensitivity 1 (n1 = 19, n2 = 51)	33.0 [27.3, 38.7]	18.0 [11.4, 24.5]	21.2 [13.6, 27.4]	29.4 [20.1, 38.7]	28.2 [17.7, 38.8]
Sensitivity 2 (n1 = 19, n2 = 48)	33.0 [27.2, 38.8]	19.2 [12.7, 25.8]	21.5 [14.6, 28.3]	30.7 [21.0, 40.4]	29.0 [18.1, 40.0]
Sensitivity 3 (n1 = 14, n2 = 33)	30.4 [23.7, 37.0]	17.7 [10.7, 24.8]	22.5 [14.7, 30.3]		

Notes. Sensitivity 1 = + days since last dose (baseline = 0); Sensitivity 2 = follow-ups ≤14; Sensitivity 3 = Sessions 0–2 only; n1 = n participants; n2 = n observations; SX, Session X.

### Adverse events

AEs were reported in 30 of 40 dosing sessions. In 24 (80%) of the 30 recorded dosing sessions, at least one AE was reported. Patients experiencing an AE typically reported more than one (M = 3.53, SD = 2.11, Min = 1, Max = 8). The most frequent AEs were fatigue (n = 20), headache (n = 16), and tearfulness (n = 15). No serious and sustained AEs were documented in clinical records. Table 2 contains all AEs recorded.

## Discussion

In this retrospective real-world study of patients with TRD, psilocybin was administered between one and four times, flexibly fitted to the course of each patient by clinical decisions. A clinically relevant improvement in depressive symptoms between baseline and after the final dosing session was observed on both self-reported and clinician-rated instruments. While AEs were reported in 80% of recorded sessions, no serious or sustained AEs were recorded in medical documentation. Interpretations of AE rates should be made with caution, as systematic assessment beyond routine documentation was incomplete. The mean reduction of 10.89 points (MADRS) and of 9.06 points (BDI) observed in this study were slightly lower than the mean reduction of 12.11 points across clinical trials on both TRD and MDD.^29^ Response and remission numbers were lower than in previous RCTs on MDD, which had up to 70% responders and roughly 50% remitting within weeks.^16^^,^^30^^,^^31^ However, they fell within the lower range of recent larger RCTs with response rates of 37–42%, and remission rates of 25–29%.^14^^,^^15^ They were also comparable to those reported in large antidepressant effectiveness trials such as STAR∗D.^3^ This likely reflects the high degree of treatment resistance and clinical complexity of patients referred for psilocybin therapy in routine care. The effect sizes found in this study were also within the range of prior studies, but smaller.^29^ Taken together, these findings indicate that results from clinical trials, to some extent, may translate into routine clinical practice, although outcomes appear smaller in scale. However, such comparisons warrant caution, as existing studies involved only one or two dosing sessions, ruled out concomitant medications, and focused mainly on MDD rather than TRD.

Across sessions, the most robust improvement was observed after the first dosing session on both scales. While improvement from baseline remained after session two, uncertainty increased towards sessions three and four, where data sparsity made it difficult to discern improvement from noise. Therefore, results for sessions three and four warrant cautious interpretation and provide little evidence for a trend beyond improvements after session one. Furthermore, results diverged across instruments, on the MADRS estimates and associated CIs remained broadly consistent with improvements similar to session one, while on the BDI they moved towards baseline. Sensitivity analyses were consistent with the main model. Early-session estimates were robust to adjustments for time since dosing, follow-up restrictions, and exclusion of patients with three or four sessions, while later-session estimates remained uncertain. Several explanations are plausible for the lack of additional observed benefit. First, there were very few data towards sessions three and four, making estimates imprecise. Second, treatment was often continued in patients with partial response, relapse, or non-response, raising the possibility of confounding by a more severe treatment resistance among those receiving >2 sessions. Third, the time between dosing sessions varied greatly between patients, and it may be that there is a specific time window in which an additional dose is most effective. There are, however, currently no data to support this claim. Finally, there might simply be no additive or even a maintaining effect of two or more psilocybin sessions. The two meta-analyses conducted on this topic yield inconsistent results; one found that two psilocybin sessions had a greater and longer-lasting effect than one, while the other failed to find a statistically robust difference.^18^^,^^29^ Importantly, our data are not suitable to draw conclusions about the optimal number or timing of sessions, and later-session findings are descriptive only. Even though we observed no further improvement at the group level, there might still be a value in multiple psilocybin sessions, as some patients responded only after the second or third session.

The study at hand is among the first to describe a multidose paradigm as well as the feasibility of psilocybin in a naturalistic clinical real-world setting. The naturalistic setting is especially important for patients with TRD, as they may not be adequately included in trials due to strict exclusion criteria.^4^ By including individuals with complex treatment resistance, multiple comorbidities, numerous unsuccessful antidepressant trials, several augmentation attempts, and chronic depression of many years’ duration, our sample captures clinical features commonly seen in tertiary TRD care.^4^ Finally, our paradigm provides unique insights into how psilocybin could be used in practice and may inform the design of future trials.

There are limitations that need to be acknowledged. A major limitation was the small sample size, which limits generalisability.^32^ This was most apparent in the widening of CIs after the third and fourth sessions on both instruments. Sample size was limited within the observation period by the resource-intensive delivery model. Further, female patients and non-European patients were underrepresented, also reducing generalisability. We did not include a control cohort, substantially limiting causal inference. Consequently, the observed improvements attributed to the psilocybin treatment could fully or partially be explained by regression to the mean and natural symptom fluctuations. Moreover, the available data were limited to the earliest post-session assessment; therefore, we could not evaluate the persistence of treatment effects, and the observed changes may have been transient. Furthermore, treatment procedures and data collection were guided by clinical practice rather than a standardised research protocol. As a result, substantial heterogeneity was present across treatment duration, psilocybin dose, number of sessions, measurement time points, concomitant psychopharmacotherapy, psychotherapy, setting, and follow-up schedules. Together with the small sample size, this introduced another major limitation: it was not feasible to statistically control for all potential confounders beyond assessment-timing. Confounding due to unmeasured or unmodeled factors therefore, cannot be ruled out and may have led to over- or underestimation of the true treatment effect. In particular, concomitant medication was managed according to clinical decisions rather than standardised protocols; therefore, their independent contribution cannot be fully disentangled. That said, most medication changes reflected temporary holds or discontinuations rather than treatment intensification, which makes it less likely that concomitant medication changes alone explain the observed symptom reductions. Relatedly, concomitant and prior serotonergic antidepressant use has been associated with attenuation of acute subjective psychedelic effects, which could have influenced the intensity of the drug experience and downstream outcomes in our cohort.^33^ Furthermore, psychotherapeutic support was not strictly manualised, varied slightly between settings, and concomitant outpatient sessions were not prohibited.^34^^,^^35^ These variabilities make it difficult to attribute changes to psilocybin alone. Therefore, clinical effect estimates should be interpreted as preliminary and may be biased upwards by expectancy effects and regression to the mean.

Future research could mitigate some of these limitations by using prospective observational designs comparing psilocybin treatment with other treatment options for TRD. Ideally, larger observational studies should control for variables such as concomitant medication and psychotherapy, setting, and treatment duration. Given the resource-intensive nature of current psilocybin treatment paradigms,^20^ future studies should aim to identify predictors for treatment success that enable patient stratification to increase effectiveness and economic viability.

In sum, this study demonstrates that psilocybin treatment was associated with clinically meaningful improvement in depressive symptoms among patients with TRD, transferring findings from clinical trials with slightly lowered clinical improvement. We further explored the course of multiple psilocybin sessions, but sparse data provided no clear signal of additive benefit. This study marks an important first step in investigating the outcome and safety of multiple, flexibly scheduled psilocybin sessions in clinical practice. In the context of emerging European compassionate-use frameworks, these data provide clinical estimates and may inform replication efforts and potential future implementation. For more definitive conclusions, studies with larger sample sizes are needed.

## Contributors

JJ: Conceptualisation, Methodology, Study Design (lead), Data Curation, Formal Analysis, Investigation, Therapeutic Intervention, Writing—Original Draft, Visualisation. SW: Data Curation, Methodology, Formal Analysis (lead), Visualisation, Writing—Original Draft (lead). HDA: Methodology, Investigation, Therapeutic Intervention, Writing—Review & Editing; BP: Investigation, Writing—Review & Editing; GK: Writing—Review & Editing; ES: Writing—Review & Editing; SP: Methodology, Investigation, Therapeutic Intervention, Writing—Review & Editing; SO: Conceptualisation, Supervision, Investigation, Therapeutic Intervention, Writing—Review & Editing.

## Data sharing statement

Deidentified individual participant data will not be shared because the dataset is small and derived from routine clinical care, limiting safe deidentification. Aggregate data underlying the main results, a data dictionary, and analysis code will be available from publication upon reasonable request to the corresponding author (johannes.jungwirth@pukzh.ch). Access is for research purposes subject to proposal review and, where applicable, a data use agreement; no identifiers will be shared.

## Declaration of use of artificial intelligence (AI)

The AI-based tool ChatGPT (model: GPT-5 Thinking, OpenAI) was used at certain points for support in translation and wording. The content, ideas, structure, argumentation, and selection of the literature originate entirely from the authors. The authors take full responsibility for the text and the work as a whole. All AI-assisted passages were reviewed, revised, and source-verified by the authors. No patient data were entered into any AI system.

## Declaration of interests

J.J. received honoraria for lectures and advisory board participation from Janssen Switzerland and honoraria for a talk from Schwabe Pharma. H.D.A. has received advisory fees from Mind Medicine Inc., Open Foundation, California Institute of Integral Studies, and honoraria from the Federal Office of Public Health and from the Swiss Medical Society for Psychedelic Therapy. B.P. received honoraria for lectures from SGIP-SSPI, Johnson&Johnson and Lundbeck. E.S. has received honoraria for educational lectures, consulting fees and unrestricted educational grants from Angelini, Boehringer Ingelheim, Lundbeck, Mepha, OM Pharma, Otsuka, Ricordati, Roche Pharma, Sandoz, Schwabe Pharma, Janssen Cilag, Servier, Sunovion, Takeda and Vifor. S.P. received honoraria for a lecture for Beltz Verlag. S.O. received honoraria for lectures and advisory board participation from Janssen Switzerland and honoraria for talks from Schwabe Pharma and Idorsia. S.O. is co-founder of DeepPSY AG. All other authors declare no conflict of interest.
