# Supplementary material for: Real-World Psilocybin Therapy for Treatment-Resistant Depression: A Retrospective Observational Study
Source: Lancet Reg Health Eur. 2026 Jun 1;67:101719. doi: 10.1016/j.lanepe.2026.101719 (PMC13453954; doi:10.1016/j.lanepe.2026.101719)
Supplement: Supplementary file 1 [file mmc1.docx]

**Supplementary information (SI)**

**Detailed description of the procedure**

| **Table S1. Patient-level exposure** | | | | | | |
| --- | --- | --- | --- | --- | --- | --- |
| **Patients** | **Dose in mg** | **Time to next session in days** | **Setting** | **Antidepressant medication status** | **Augmentation medication status** | **Relevant concomitant medication** |
| **Session 1** | | | | | | |
| Patient 2 | 25 | 13 | Inpatient group | **Sertraline:** stopped 5 days before dosing; not restarted. | **Quetiapine:** stopped 5 days before dosing; not restarted. | None |
| Patient 3 | 25 | / | Inpatient single | **Escitalopram:** paused on dosing day only. | None  **Change from baseline only:** Quetiapine had been stopped outside the measurement period. | None |
| Patient 4 | 20 | 69 | Inpatient single | **Escitalopram:** stopped 7 days before dosing session 1; not restarted | None | **Mirtazapine 7.5 mg:** taken once 5 days before dosing as sleep medication. **Trimipramine 25 mg, trazodone 50 mg, and quetiapine 25 mg:** each taken once 3 days before dosing as sleep medication. |
| Patient 5 | 30 | 70 | Inpatient single | **Vortioxetine:** tapered to 10 mg and then fully paused 2 weeks before dosing session 1; restarted at 20 mg 1 week later. | None | None |
| Patient 6 | 25 | 15 | Inpatient group | **Agomelatine:** paused on dosing day only.  **Change from baseline only:** Moclobemide and Melitracen had been stopped outside the measurement period. | None | **Alprazolam:** taken once 3 days before dosing and once 3 days after dosing.  **Daridorexant:** paused on dosing day. |
| Patient 7 | 25 | 15 | Inpatient group | **Bupropion:** paused on dosing day only.  **Trazodone 150mg:** stopped 4 days before dosing; not restarted. | None | None |
| Patient 8 | 25 | 72 | Inpatient single | **Fluoxetine:** paused 1 week before dosing; restarted 3 days after treatment. | None  **Change from baseline only:** Quetiapine had been stopped outside the measurement period. | **Alprazolam:** taken once 2 days before dosing. |
| Patient 9 | 25 | 14 | Inpatient group | **Duloxetine:** paused on dosing day only. | None  **Change from baseline only:** Quetiapine had been stopped outside the measurement period. | **Trazodone 75mg:** as sleep medication, paused 4 days before dosing. |
| Patient 10 | 25 | 14 | Inpatient group | **Duloxetine:** paused on dosing day only.  **Trazodone 150mg:** paused 1 month before dosing (outside of measurement period). | **Quetiapine:** paused 6 days before dosing; resumed 1 day after dosing. | **Alprazolam:** taken daily concomitantly. |
| Patient 11 | 25 | / | Inpatient group | **Venlafaxine:** paused 6 days before dosing; resumed the next day. | None | **Lorazepam:** taken 2 days before dosing and 1 day after dosing.  **Pregabalin:** paused on dosing day.  **Trazodone 50mg:** as sleep mediaction, paused 2 days before dosing.  **Quetiapine:** as sleep medication, taken once 3 days before dosing. |
| Patient 12 | 25 | 78 | Inpatient single | **Venlafaxine:** paused on dosing day and stopped after treatment.  **Trazodone 150mg:** stopped 5 days before dosing. | None | None  **Clonazepam:** had been stopped before dosing, outside the measurement period. |
| Patient 13 | 25 | 14 | Inpatient group | **Venlafaxine:** paused on dosing day only. | None | **Clonazepam 1 mg/day:** continued concomitantly.  **Daridorexant:** started 1 week after dosing session 1. |
| Patient 14 | 25 | 14 | Inpatient group | **Duloxetine:** paused on dosing day only. | **Lithium:** paused 2 weeks before dosing session 1; restarted 1 day after dosing session 2. | None |
| Patient 15 | 30 | / | Inpatient single | None | None | None |
| Patient 16 | 25 | / | Inpatient group | **Vortioxetine:** paused on dosing day only. | None | **Quetiapine:** as sleep mediaction, taken once 5 days before dosing.  **Daridorexant:** continued concomitantly, including on the evening of the treatment day. |
| Patient 17 | 25 | 14 | Inpatient group | **Venlafaxine:** paused on dosing day only. | None | None |
| Patient 18 | 25 | 14 | Inpatient group | **Fluoxetine:** paused on dosing day only. | **Aripiprazole:** paused 2 days before dosing after tapering over the preceding 7 days; replaced with **quetiapine** 22 days after session 2.  **Lithium:** stopped 7 days before dosing and not restarted. | None |
| Patient 19 | 25 | / | Outpatient single | None | None | None |
| Patient 20 | 25 | / | Inpatient Single | **Escitalopram:** paused on dosing day only.  **Trazodone 200mg:** paused 4 days before dosing. | None | **Lorazepam:** taken once 1 day after the session. |
| **Session 2** | | | | | | |
| Patient 2 | 25 | 71 | Inpatient single | None | None | None |
| Patient 3 | / | / | / | / | / | / |
| Patient 4 | 30 | 182 | Inpatient single | None | None | None |
| Patient 5 | 35 | / | Inpatient single | **Vortioxetine:** stopped 2 weeks before dosing session 2; restarted 2 weeks later (outside the measurement period). | None | None |
| Patient 6 | 30 | 84 | Inpatient group | **Agomelatine:** paused on dosing day only. | None | **Alprazolam:** taken 4 days before dosing.  **Daridorexant:** paused on dosing day. |
| Patient 7 | 30 | / | Inpatient group | **Bupropion:** paused 3 days before dosing. | None | None |
| Patient 8 | 25 | 250 | Inpatient single | **Fluoxetine:** paused on dosing day only. | None | **Lorazepam:** taken once 1 day after the session.  **Methylphenidate:** paused on dosing day. |
| Patient 9 | 25 | / | Inpatient group | **Duloxetine:** paused on dosing day only. | None | **Trazodone 75mg:** as sleep medication, paused 4 days before dosing. |
| Patient 10 | 25 | 84 | Inpatient group | **Duloxetine:** paused on dosing day only.  **Trazodone 150mg:** had been paused more than 30 days before dosing; resumed 6 days after dosing. | **Quetiapine:** paused 5 days before dosing. | **Alprazolam:** continued on dosing day and not paused.  **Quetiapine:** as sleep medication, taken 1 day before dosing. |
| Patient 11 | / | / | / | / | / | / |
| Patient 12 | 25 | / | Inpatient single | None | None | None |
| Patient 13 | 25 | / | Inpatient group | **Venlafaxine:** paused on dosing day only.  **Trazodone 150 mg:** started 1 day after dosing session 2. | None | **Clonazepam 1 mg/day:** continued concomitantly.  **Daridorexant 25 mg:** started 1 week before dosing session 2 and stopped 2 days afterward. |
| Patient 14 | 30 | / | Inpatient group | **Duloxetine 90 mg:** paused on dosing day only. | **Lithium:** remained paused after dosing session 1 and was resumed 1 day after dosing session 2. | None |
| Patient 15 | / | / | / | / | / |  |
| Patient 16 | / | / | / | / | / |  |
| Patient 17 | 30 | / | Inpatient group | **Venlafaxine:** paused on dosing day only. | None | None |
| Patient 18 | 30 | / | Inpatient group | **Fluoxetine:** continued on dosing day; not paused. | None | None |
| Patient 19 | / | / | / | / | / | / |
| Patient 20 | / | / | / | / | / | / |
| **Session 3** | | | | | | |
| Patient 2 | 25 | 14 | Inpatient group | None | None | None |
| Patient 3 | / | / | / | / | / |  |
| Patient 4 | 35 | / | Inpatient single | None | None | None |
| Patient 5 | / | / | / | / | / |  |
| Patient 6 | 30 | 14 | Inpatient  group | **Agomelatine:** paused on dosing day only. | None | **Lorazepam:** taken once 1 day after dosing.  **Daridorexant:** paused on dosing day. |
| Patient 7 | / | / | / | / | / |  |
| Patient 8 | 25 | / | Inpatient  single | **Treatment changed to**  **Vortioxetine:** paused on dosing day only. | None | **Methylphenidate:** paused on dosing day. |
| Patient 9 | / | / | / | / | / |  |
| Patient 10 | 25 | 14 | Inpatient group | **Duloxetine:** paused on dosing day only.  **Trazodone 150mg:** paused 5 days before dosing, resumed 3 days after | **Quetiapine:** paused 3 days before dosing. | **Alprazolam:** paused on dosing day. |
| Patient 11 | / | / | / | / | / |  |
| Patient 12 | / | / | / | / | / |  |
| Patient 13 | / | / | / | / | / |  |
| Patient 14 | / | / | / | / | / |  |
| Patient 15 | / | / | / | / | / |  |
| Patient 16 | / | / | / | / | / |  |
| Patient 17 | / | / | / | / | / |  |
| Patient 18 | / | / | / | / | / |  |
| Patient 19 | / | / | / | / | / |  |
| Patient 20 | / | / | / | / | / |  |
| **Session 4** | | | | | | |
| Patient 2 | 25 | / | Inpatient group | None | None | **Mirtazapine 15mg:** as sleep medication, used once 5 days before session 4. |
| Patient 3 | / | / | / | / | / |  |
| Patient 4 | / | / | / | / | / |  |
| Patient 5 | / | / | / | / | / |  |
| Patient 6 | 30 | / | Inpatient group | **Agomelatine:** paused on dosing day only. | None | **Trazodone 75mg:** as sleep medication, started 2 days after session 4.  **Mirtazapine 15mg:** as sleep medication, used 5 days before and 1 day after the dosing session 4, then discontinued.  **Daridorexant:** discontinued before treatment, restarted 20 days afterwards, and fully stopped again 1 week afterward. |
| Patient 7 | / | / | / | / | / |  |
| Patient 8 | / | / | / | / | / |  |
| Patient 9 | / | / | / | / | / |  |
| Patient 10 | 25 | / | Inpatient group | **Escitalopram:** switched after session 3; paused on dosing day.  **Trazodone 150mg:** paused 19 days before dosing. | **Quetiapine:** paused 3 days before dosing. | **Alprazolam:** paused on dosing day. |
| Patient 11 | / | / | / | / | / | / |
| Patient 12 | / | / | / | / | / | / |
| Patient 13 | / | / | / | / | / | / |
| Patient 14 | / | / | / | / | / | / |
| Patient 15 | / | / | / | / | / | / |
| Patient 16 | / | / | / | / | / | / |
| Patient 17 | / | / | / | / | / | / |
| Patient 18 | / | / | / | / | / | / |
| Patient 19 | / | / | / | / | / | / |
| Patient 20 | / | / | / | / | / | / |
| Legend: Patients were namend starting with Patient 2, as Patient 1 was not included, *None* refers to no relevant concomitant medication at that timepoint in that category; | | | | | | |

Medication management across sessions

Antidepressant and concomitant medication status across sessions was structured as follows. At dosing session 1 (*n* = 19), two patients were without antidepressant medication, 14 paused medication but otherwise continued the same regimen, and three stopped medication before or after the dosing day. Of these 19 patients, six received an atypical antipsychotic (3 paused and 3 stopped before the dosing day) and two augmentation with lithium (both paused or stopped 1-2 weeks prior to the dosing day). Six patients took mirtazapine or trazodone (all paused or stopped prior to dosing), two patients were on benzodiazepines, and four took benzodiazepines once around the dosing day. At dosing session 2 (*n* = 13), three patients had no antidepressant medication, seven paused medication but otherwise continued the same regimen, one remained on antidepressant medication on the dosing day, one stopped antidepressant medication prior to session 2, and one changed treatment. Of these 13 patients, two received an atypical antipsychotic or augmentation with lithium (both paused before dosing session). Two patients took trazodone (all paused before dosing session), one patient was on benzodiazepines, and two took benzodiazepines once around dosing session 2. At dosing session 3 (*n* = 5), two patients were without antidepressant medication, two paused medication but otherwise continued the same regimen, and one changed antidepressant medication. Of these five patients, one received an atypical antipsychotic (paused before dosing session). One patient took trazodone (paused before dosing session), one patient was on benzodiazepines, and two took benzodiazepines once around dosing session 3. At dosing session 4 (*n* = 3), one patient remained without antidepressant medication, one paused medication but otherwise continued the same regimen, and one changed antidepressant medication. One patient had trazodone paused prior to session 4, in one patient trazodone was initiated after session 4, and one patient took benzodiazepines (paused on treatment day).

Preparation

At least 8 weeks prior to the first dosing session, an indication meeting was held. The psychiatric and biographical history, psychopathology, diagnosis, prior treatment trials, indication, contraindication, and medication history were assessed. BDI and MADRS were conducted. Further, an EEG, ECG, blood test, and clinical cMRI were required. Apart from these objective measures, the patient’s expectations, goals, and the potential for establishing a sustainable working alliance were assessed. Patients received thorough information on the expected acute effects, possible AEs, the potential mechanisms of action, and gave written informed consent to the treatment. At least 2 preparation meetings where held within 1-14 days prior to the dosing session. The primary objective of these meetings was to build a therapeutic relationship between the clinical staff present at the dosing session and the patient. Furthermore, they were used to address any questions and fears the patient might have, as well as their expectations and intentions for the upcoming treatment. Patients were encouraged to set specific goals for behavioral changes. In the inpatient group setting, patients had group preparation meetings to further build an agreeable and trustworthy group environment.

Medication management

Prior to the administration of psilocybin, concomitant medications were reviewed. Augmentation medication with antipsychotics was discontinued 1-2 weeks prior to treatment, due to their attenuating effect on psychedelics. Antidepressant medication was generally paused in the morning of the dosing day, full discontinuation was considered on a case by case basis. Trazodone and other medication with 5-HT2 receptor blocking properties were paused at least three days prior to the dosing session. Monoamine oxidase inhibitors and lithium were paused or stopped prior to treatment. It was recommended to wean off benzodiazepines or at least reduce the dose. The effects of other concomitant medications are not well studied, temporary discontinuation on the day of treatment was considered when appropriate.

Dosing session

The dosing day for patients in the single setting as well as the inpatient group settings began with a repetition of helpful behaviors such as: acceptance of the experience, introspection, breathing exercises and management of difficult emotions. The session took place in a quiet, comfortable and specifically arranged room. Patients were lying down on a bed, sofa or mattress, equipped with a sleep mask, cushions and a blanket. Music was played via headphones or loudspeakers. The choice of music was specifically tailored for this treatment and was meant to facilitate a comfortable but also emotionally engaging experience.

Follow-up and integration

In the single setting, after the dosing sessions patients had at least two in most cases three integration meetings. The first was held 1-2 days after the dosing session. It was primarily used to thoroughly describe and retell the experience. Potential first insights were discussed. The next integration meeting was scheduled 7-14 days after the dosing session. This meeting was used to discuss potential progress, changes and insights that had emerged since dosing. Specifically, the meaning of the experience for day to day life was discussed. In the third and final integration meeting scheduled 14-30 days after the treatment, the past few weeks after the treatment were discussed, as well as current subjective well-being. If needed, next treatment steps were discussed. On at least one of these three occasions, MADRS and BDI were conducted.

In the inpatient group setting, the content of the follow-up meetings stayed the same. The setting was, however, slightly different. After the first dosing session, they had at least three integration sessions, two of which were held in groups. Along these integration sessions, patients were participating in the basic psychiatric treatment procedures of the ward. After the second dosing session, patients had at least one more integration session 1-3 days after the second dosing session. MADRS and BDI were most often only conducted during the final integration session after the second dosing session.

**Supplementary information on statistical analysis**

R-Packages

Tidyverse (version 2.0.0)**,** patchwork (version 1.3.2), lme4 (version 1.1-37), sjPlot (version 2.9.0), performance (version 0.15.2), lmtest (version 0.9-40), emmeans (2.0.0), car (version 3.1-3)**,** influence.ME (version 0.9-9), lattice (version 0.22-7), officer (version 0.7.0), flextable (version 0.9.10), janitor (version 2.2.1).

Assumption testing

Linear mixed-effects model

Normality of the residuals was assessed by inspection of residual Q-Q plots. No unusual observations across analyses. Normality of the random effect was assessed by inspecting the Q–Q plot of the estimated random effects. No unusual observations across analyses. Homoscedasticity was assessed by plotting residuals grouped by session to verify approximately constant variance across levels. No unusual observations across analyses. Influential points were identified using cooks' distance and applied on the group level using the N divided by 4 rule. Influential patients did not influence the results in a meaningful way. The following extra assessments were made for the model containing days since dosing session (Sensitivity 1). On both the MADRS and BDI, residuals plotted against days since dosing session showed no clear systematic nonlinearity. Deviations at very short delays post session were considered smoothing artefacts given clustering of observations at low values and sparse data at longer follow-up delays.

**Supplementary results**

Sensitivity primary endpoint analyses

| **Table S2: Results sensitivity endpoint analyses** | | | | | | |
| --- | --- | --- | --- | --- | --- | --- |
|  | **MADRS** | | | **BDI** | | |
| Predictors | b | SE | 95% CI | b | SE | 95% CI |
| Intercept | -2.68 | 6.2 | -16.0, 10.6 | -0.9 | 7.6 | -17.1, 15.2 |
| Pre score | .67 | .12 | 0.3, 1.1 | 0.7 | 0.2 | .24, 1.1 |
| Days since dose | .16 | .20 | -0.1, 0.4 | 0.2 | 0.2 | -0.3, 0.7 |
| Notes. The same model was refitted with days since dosing session recalculated to weeks since dosing session to derive 95% CI for the 7 day change. | | | | | | |

Primary: linear mixed-effects model

| **Table S3a Primary: linear mixed-effects model** | | | | | | |
| --- | --- | --- | --- | --- | --- | --- |
|  | ***MADRS*** | | | ***BDI*** | | |
| Fixed Effect Structure | | | | | | |
| Predictors | b | SE | 95% CI | b | SE | 95% CI |
| Intercept | 31.32 | 2.17 | 26.94, 35.42 | 33.00 | 2.95 | 26.62, 38.92 |
| Session 1 | -13.06 | 2.39 | -17.75, - 8.25 | -12.42 | 2.72 | -17.96, -7.28 |
| Session 2 | -9.22 | 2.46 | -14.20, -4.41 | -9.13 | 2.80 | -14.62, -3.09 |
| Session 3 | -10.86 | 3.87 | -18.49, -3.43 | -3.25 | 4.51 | -12.20, 5.62 |
| Session 4 | -15.17 | 5.32 | -25.76, -4.62 | -5.49 | 5.17 | -15.48, 5.28 |
| Random Effects Structure | | | | | | |
| σ^2^ | 41.25 | | | 52.61 | | |
| τ_00_ | 47.82 | | | 112.35 | | |
| ICC | 0.54 | | | 0.68 | | |
| *N* | 19 | | | 19 | | |
| Marginal *R^2^* / Conditional *R^2^* | 0.276 / 0.665 | | | 0.142 / 0.726 | | |

| **Table S3b Primary: Estimated marginal means** | | | | | | | | |
| --- | --- | --- | --- | --- | --- | --- | --- | --- |
|  | **MADRS** | | | | **BDI** | | | |
| Predictors | Estimate | *SE* | *df* | 95% CI | Estimate | *SE* | *df* | 95% CI |
| Baseline | 31.3 | 2.17 | 30.9 | [26.9, 35.7] | 33.0 | 2.95 | 26.3 | [26.9, 39.1] |
| Session 1 | 18.3 | 2.47 | 38.9 | [13.3, 23.3] | 20.6 | 3.25 | 33.8 | [14, 27.2] |
| Session 2 | 22.1 | 2.55 | 40.3 | [17, 27.2] | 23.9 | 3.32 | 35.4 | [17.1, 30.6] |
| Session 3 | 20.5 | 3.96 | 42.6 | [12.5, 28.4] | 29.8 | 4.89 | 45.6 | [19.9, 39.6] |
| Session 4 | 16.1 | 5.42 | 38.0 | [5.2, 27.1] | 27.5 | 5.52 | 44.1 | [16.4, 38.6] |

Sensitivity: linear mixed effects model

Sensitivity 1:

| **Table S4a Sensitivity 1: linear mixed-effects model** | | | | | | |
| --- | --- | --- | --- | --- | --- | --- |
|  | ***MADRS*** | | | ***BDI*** | | |
| Fixed Effect Structure | | | | | | |
| Predictors | b | SE | 95% CI | b | SE | 95% CI |
| Intercept | 31.32 | 2.05 | 27.17, 35.46 | 33.00 | 2.77 | 27.42, 38.58 |
| Session 1 | -15.86 | 2.61 | -21.12, -10.60 | -15.02 | 2.82 | -20.71, -9.33 |
| Session 2 | -13.11 | 2.93 | -19.02, -7.20 | -12.49 | 3.03 | -18.60, -6.38 |
| Session 3 | -12.29 | 3.78 | -19.93, -4.66 | -3.57 | 4.34 | -12.33, 5.18 |
| Session 4 | -14.71 | 5.12 | -25.05, -4.37 | -4.78 | 4.97 | -14.80, 5.25 |
| Days since dose | 0.26 | 0.11 | 0.03, 0.48 | 0.36 | 0.15 | 0.06, 0.66 |
| Random Effects Structure | | | | | | |
| σ^2^ | 38.41 | | | 48.73 | | |
| τ_00_ _id_ | 41.79 | | | 96.87 | | |
| ICC | 0.52 | | | 0.67 | | |
| N _id_ | 19 | | | 19 | | |
| Observations | 50 | | | 51 | | |
| Marginal R^2^ / Conditional R^2^ | 0.336 / 0.682 | | | 0.211 / 0.736 | | |

| **Table S4b Sensitivity 1: Estimated marginal means** | | | | | | | | |
| --- | --- | --- | --- | --- | --- | --- | --- | --- |
|  | **MADRS** | | | | **BDI** | | | |
| Predictors | Estimate | *SE* | *df* | 95% CI | Estimate | *SE* | *df* | 95% CI |
| Baseline | 31.3 | 2.05 | 30.76 | 27.12, 35.51 | 33.0 | 2.77 | 26.39 | 27.31, 38.69 |
| Session 1 | 15.5 | 2.64 | 41.92 | 10.12, 20.79 | 18.0 | 3.24 | 36.95 | 11.42, 24.54 |
| Session 2 | 18.2 | 2.96 | 43.48 | 12.24, 24.17 | 20.5 | 3.42 | 40.14 | 13.59, 27.43 |
| Session 3 | 19.0 | 3.81 | 41.35 | 11.33, 26.71 | 29.4 | 4.62 | 44.35 | 20.11, 38.74 |
| Session 4 | 16.6 | 5.14 | 36.82 | 6.18, 27.02 | 28.2 | 5.22 | 42.77 | 17.69, 38.75 |

Sensitivity 2:

| **Table S5a Sensitivity 2: linear mixed-effects model** | | | | | | |
| --- | --- | --- | --- | --- | --- | --- |
|  | ***MADRS*** | | | ***BDI*** | | |
| Fixed Effect Structure | | | | | | |
| Predictors | b | SE | 95% CI | b | SE | 95% CI |
| Intercept | 31.32 | 2.00 | 27.27, 35.36 | 33.00 | 2.82 | 27.30, 38.70 |
| Session 1 | -13.13 | 2.51 | -18.22, -8.03 | -13.78 | 2.79 | -19.42, -8.14 |
| Session 2 | -12.98 | 2.85 | -18.76, -7.20 | -11.52 | 2.99 | -17.55, -5.49 |
| Session 3 | -10.20 | 3.83 | -17.95, -2.45 | -2.29 | 4.52 | -11.41, 6.83 |
| Session 4 | -12.97 | 5.29 | -23.68, -2.25 | -3.96 | 5.19 | -14.44, 6.53 |
| Random Effects Structure | | | | | | |
| σ^2^ | 40.00 | | | 51.59 | | |
| τ_00_ _id_ | 35.65 | | | 99.97 | | |
| ICC | 0.47 | | | 0.66 | | |
| N _id_ | 19 | | | 19 | | |
| Observations | 44 | | | 48 | | |
| Marginal R^2^ / Conditional R^2^ | 0.349 / 0.656 | | | 0.200 / 0.728 | | |

| **Table S5b Sensitivity 2: Estimated marginal means** | | | | | | | | |
| --- | --- | --- | --- | --- | --- | --- | --- | --- |
|  | **MADRS** | | | | **BDI** | | | |
| Predictors | Estimate | *SE* | *df* | 95% CI | Estimate | *SE* | *df* | 95% CI |
| Baseline | 31.3 | 2.00 | 29.22 | 27.24, 35.40 | 33.0 | 2.82 | 26.38 | 27.20, 38.80 |
| Session 1 | 18.2 | 2.47 | 37.65 | 13.19, 23.19 | 19.2 | 3.22 | 35.16 | 12.69, 25.75 |
| Session 2 | 18.3 | 2.81 | 39.00 | 12.65, 24.03 | 21.5 | 3.39 | 38.25 | 14.62, 28.33 |
| Session 3 | 21.1 | 3.80 | 35.64 | 13.42, 28.82 | 30.7 | 4.79 | 42.24 | 21.05, 40.38 |
| Session 4 | 18.3 | 5.27 | 32.07 | 7.63, 29.07 | 29.0 | 5.43 | 40.68 | 18.07, 40.02 |

Sensitivity 3:

| **Table S6a Sensitivity 3: linear mixed-effects model** | | | | | | |
| --- | --- | --- | --- | --- | --- | --- |
|  | ***MADRS*** | | | ***BDI*** | | |
| Fixed Effect Structure | | | | | | |
| Predictors | b | SE | 95% CI | b | SE | 95% CI |
| Intercept | 30.86 | 2.40 | 25.94, 35.77 | 30.36 | 3.18 | 23.84, 36.87 |
| Session 1 | -15.07 | 2.18 | -19.54, -10.60 | -12.61 | 3.19 | -19.14, -6.08 |
| Session 2 | -8.09 | 2.39 | -12.99, -3.19 | -7.87 | 3.61 | -15.26, -0.48 |
| Random Effects Structure | | | | | | |
| σ^2^ | 40.00 | | | 59.68 | | |
| τ_00_ _id_ | 35.65 | | | 82.02 | | |
| ICC | 0.47 | | | 0.58 | | |
| N _id_ | 19 | | | 14 | | |
| Observations | 44 | | | 33 | | |
| Marginal R^2^ / Conditional R^2^ | 0.349 / 0.656 | | | 0.182 / 0.656 | | |

| **Table S6b Sensitivity 3: Estimated marginal means** | | | | | | | | |
| --- | --- | --- | --- | --- | --- | --- | --- | --- |
|  | **MADRS** | | | | **BDI** | | | |
| Predictors | Estimate | *SE* | *df* | 95% CI | Estimate | *SE* | *df* | 95% CI |
| Baseline | 30.8 | 2.40 | 18.47 | 25.83, 35.88 | 30.36 | 3.18 | 21.27 | 23.75, 36.97 |
| Session 1 | 15.8 | 2.61 | 22.78 | 10.38, 21.20 | 17.7 | 3.43 | 24.63 | 10.68, 24.82 |
| Session 2 | 22.8 | 2.79 | 25.47 | 17.03, 28.50 | 22.5 | 3.82 | 28.15 | 14.65, 30.31 |

| **Table S8 Management of concomitant medications for psilocybin, with emphasis on agents affecting 5-HT2A signaling** | | |
| --- | --- | --- |
| **Medication class** | **Recommendation** | **Relevance to 5-HT2A signaling** |
| Antipsychotics | Pause ≥3 days prior (reduced overall effect; potentially increased anxiety/bad drug effects with D2 antagonists) | Direct/strong antagonism → prioritized for pausing |
| Mirtazapine | Pause 5–7 days prior | 5-HT2A antagonistic properties → pause when feasible |
| Trazodone | Pause 1–2 days prior | 5-HT2A antagonistic properties → pause when feasible |
| SSRIs / SNRIs | Maintain; optional pause on treatment day | Potential attenuation reported; not primary antagonists |
| MAOI | Pause ≥3 days prior |  |
| Bupropion | Maintain | No meaningful 5-HT2A blockade |
| Lithium | Pause ≥3 days prior; possible increased seizure risk | Safety consideration rather than receptor blockade |
| Pregabalin | Maintain | No relevant 5-HT2A effect |
| Antiepileptics | Maintain |  |
| Opioids | Maintain |  |
| Benzodiazepines | Maintain; reduce dose if possible | May dampen subjective effects but not via 5-HT2A |
| Disulfiram | Pause 3 days | Interaction precaution |
| Naltrexone | Pause 1–3 days | Interaction precaution |
| Methylphenidate | Pause on treatment day | Stimulant safety consideration |
| (Lis)dexamphetamine | Pause on treatment day |  |
| Antihypertensives | Maintain | Cardiovascular safety |
| Note. This table summarizes concomitant medication management suggestions for psilocybin sessions in routine clinical care informed by the interaction table (https://saept.ch/beschraenkte-medizinische-anwendung/). Particular attention was given to medications with 5-HT2A antagonistic properties, as these agents may attenuate psychedelic effects. Such medications were systematically reviewed prior to treatment and, where clinically feasible and safe, paused in advance of dosing. All medication decisions were individualized and made collaboratively with participants to balance safety, clinical stability, and potential drug–drug interactions. | | |
